# Adenovirus Serotype 14 Infection, New Brunswick, Canada, 2011

**DOI:** 10.3201/eid1901.120423

**Published:** 2013-01

**Authors:** Gabriel Girouard, Richard Garceau, Louise Thibault, Youcef Oussedik, Nathalie Bastien, Yan Li

**Affiliations:** Author affiliations: Centre hospitalier universitaire Dr-Georges-L.-Dumont, Moncton, New Brunswick, Canada (G. Girouard, R. Garceau, L. Thibault, Y. Oussedik);; Public Health Agency of Canada, Winnipeg, Manitoba, Canada (N. Bastien, Y Li)

**Keywords:** adenovirus serotype 14, adenovirus serotype 14p1, HAdV-14p1, fatal, New Brunswick, Canada, viruses, pneumonia, respiratory infections

## Abstract

We describe 3 culture-proven cases of adenovirus serotype 14 infection in New Brunswick, Canada, during the summer of 2011. Strains isolated from severely ill patients were closely related to strains of a genomic variant, adenovirus 14p1, circulating in the United States and Ireland. Physicians in Canada should be aware of this emerging adenovirus.

Originally discovered in 1955, human adenovirus serotype 14 (HAdV-14) had rarely been reported in medical literature for over 30 years. This archetype strain, known as “agent de Wit,” seemed to have almost vanished from Earth and had not been identified in the Western Hemisphere (*1*). Since 2005, however, the number of reports of a newly emerging HAdV-14 strain has increased, mainly across the United States (*2*), and in 2009, cases were identified in Ireland (*3*). This strain, currently designated as HAdV-14p1, was confirmed by enzymatic restrictions profiles as a new genomic variant, and it has a unique signature 6-nt deletion in the knob region of the fiber gene (*4*). Outbreaks among communities and military training camps have been described, all showing high rates of infection and increased risk for hospitalization and death (*5*,*6*). It is not known whether this new circulating variant is truly more virulent or whether current reports represent only the severe side of the natural clinical spectrum of HAdV-14 in immunologically naive populations (*7*).

## The Study

In July 2011, a 74-year-old aboriginal (Micmac) woman from eastern New Brunswick sought medical care at our hospital for an influenza-like illness, including a dry cough and acute diarrhea, of 3 days’ duration. Her medical history revealed heavy smoking but was otherwise unremarkable. Chest radiographs obtained at admission revealed bilateral alveolar infiltrates. Paraclinical data for the patient during hospitalization are shown in the Table. Broad-spectrum antimicrobial drug treatment for community-acquired bacterial pneumonia was started, but the patient’s respiratory condition deteriorated rapidly. On day 1, she was transferred to the intensive care unit and intubated. Severe acute respiratory distress syndrome developed in the patient, and she died 6 days after admission. 

**Table:** Laboratory test results for a patient with a fatal case of human adenovirus-14–associated pneumonia, New Brunswick, Canada, 2011*

Laboratory test, value	Hospitalization day				Reference range
	Admission	3	5	7	
Cell count, × 10^9^/L					
Leukocytes	6.5	5.8	5.5	6.7	4.0–11.0
Polymorphonuclear cells	5.6	3.8	4.3	5.0	1.8–7.7
Lymphocytes	0.7	0.6	0.9	0.3	1.0–4.8
Platelets, × 10^9^/L	111	104	125	134	130–400
Hematocrit	0.368	0.336	0.236	0.256	0.370–0.470
PaO_2_/FiO_2_	ND	146	60	34	ND
Natremia, mmol/L	137	141	133	137	136–145
Creatinine, μmol/L	224	115	241	ND	46–92
Aspartate aminotransferase, U/L	132	63	104	55	14–36
Lactate dehydrogenase, U/L	885	1,138	1,398	1,020	313–618

*PaO_2_, arterial oxygen tension; FiO_2_, fraction of inspired oxygen; ND, no data available.

Results for PCR of nasopharyngeal and tracheal aspiration samples were negative for influenza. Results were also negative for urinary antigen detection for pneumococcus and *Legionella* spp., hantavirus IgM, and tracheal and blood cultures. Gram staining of the respiratory specimen was notable for its abundance of leukocytes and absence of bacteria. Respiratory viral cultures revealed a cytopathic effect after 6 days of incubation, and adenovirus was confirmed by indirect immunofluorescence (Light Diagnostics Respiratory Panel I Viral Screening and Identification IFA; Millipore, Billerica, MA, USA). 

**Figure 1:**
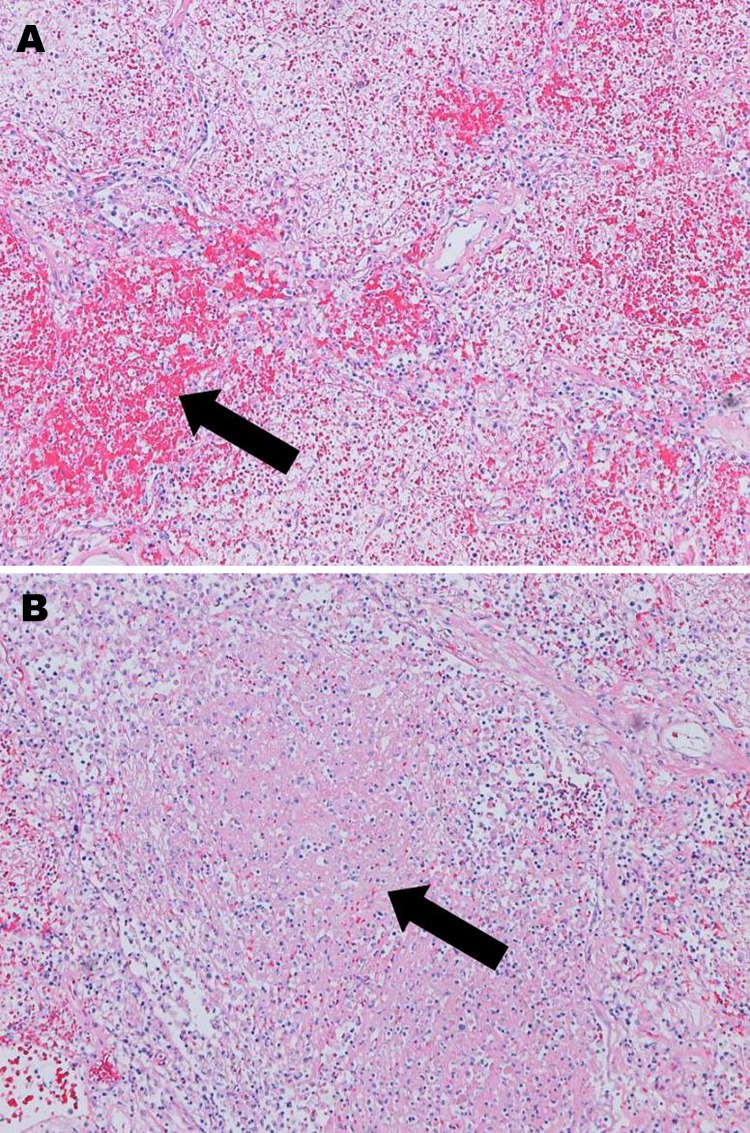
Hematoxylin and eosin–stained lung tissue (original magnification ×10) from a patient in New Brunswick, Canada, with a fatal case of pneumonia caused by adenovirus 14. A) Arrow shows a patchy area of hemorrhagic consolidation. B) Arrow shows an area of necrosis.

Given the severity of this case, the virus isolate from the patient was sent to the National Microbiology Laboratory in Winnipeg, Manitoba, Canada, to determine its serotype by partial hexon gene sequencing, as described (*8*); the infecting strain was found to be HAdV-14. Postmortem examination of the patient revealed dark purple, heavy lungs with marked consolidation and patchy hemorrhagic foci; the right lung weighed 1,593 g and the left 1,268 g (reference range 350–450 g). Examination by microscopy showed diffuse alveolar damage, fibrinohemorrhagic changes, areas of necrosis, and cellular debris (Figure 1). Immunohistochemistry (Anti-Adenovirus Antibody, clone 20/11 | MAB8052; Millipore) for adenovirus showed few epithelial cells which had cytoplasmic granular and nuclear positivity.

Once HAdV-14 had been isolated, we conducted a retrospective study of all adenovirus-positive viral cultures performed at the Provincial Laboratory for Viral Illnesses, Centre hospitalier universitaire Dr-Georges-L.-Dumont (Moncton, New Brunswick, Canada), to search for other HAdV-14 cases that might have occurred during March 21, 2011–August 31, 2011. All 14 single-patient, adenovirus-positive isolates found during the survey were submitted for partial hexon gene sequencing; HAdV-14 was found in 2 of the isolates. Of the 12 non–HAdV-14 isolates, 1 was HAdV serotype 1, 8 were HAdV serotype 2, and 3 were HAdV serotype 3. The clinical data for the 2 HAdV-14 cases was limited. The first was a case of upper respiratory tract infection and acute bilateral otitis media in a 1-year-old boy who was hospitalized in July 2011. The second was a case of severe pneumonia in a 45-year-old man who was hospitalized in August 2011. Both patients survived.

The 3 cases of HAdV-14 infection were severe, requiring hospitalization of the patients. Pneumonia developed in 2 of the patients, 1 of whom died. The cases occurred within a brief 43-day period, and the young boy and man lived within a 35-km radius of the female patient who died. The clinical outcome for these cases is consistent with previously reported clinical data on HAdV-14 infection and the apparently enhanced virulence of this strain.

**Figure 2:**
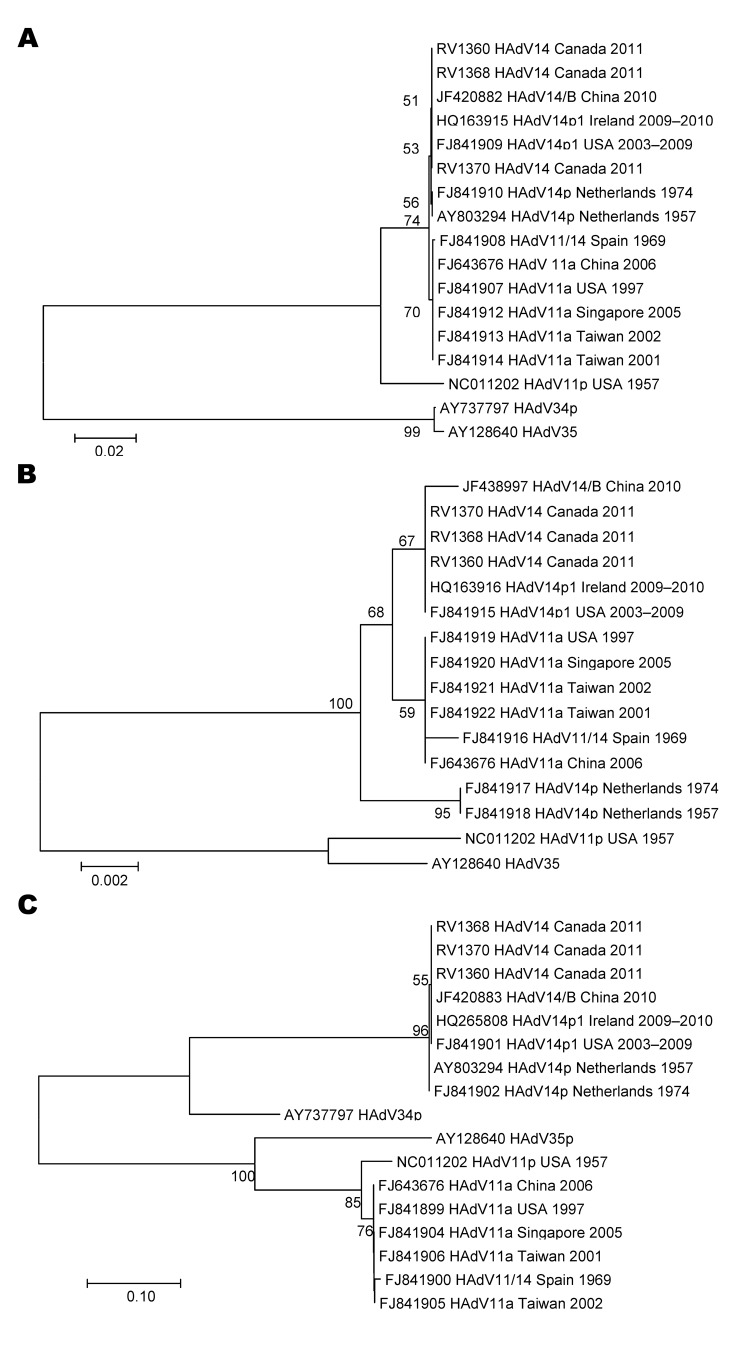
Phylogenetic analysis of human adenovirus 14 (HAdV-14) isolates from patients in New Brunswick, Canada, 2011. Nucleotide sequences were determined for the fiber (A), E1A (B), and hexon (C) genes. The corresponding gene sequences from previously reported HAdV-14 isolates are also included. Phylogenetic analysis was performed by using the neighbor-joining method of the MEGA2 program (*9*). Scale bars indicate nucleotide substitutions per site. Numbers on branches and at nodes indicate bootstrap proportions. The GenBank accession numbers for the fiber, E1A, and hexon genes for HAdV-14 (Canada) RV1360 are JQ815083, JQ815080, JQ815086, respectively; those for RV1368 are JQ815084, JQ815081, JQ815087, respectively; and those for RV1370 are JQ815085, JQ815082, JQ815088, respectively.

We performed nucleotide sequencing of the E1A, fiber, and hexon genes of the HAdV-14 isolates from Canada and compared the sequences with those for published adenovirus subspecies B2 (type 11, 14, 34, and 35) (Figure 2). We found no differences in the nucleic acid sequences of the 3 isolates from Canada, which were also identical to HAdV-14p1 strains from the United States and Ireland. The fiber gene sequence of the isolates from Canada contained the 6-nt deletion identified in HAdV-14p1 strain from the United States and Ireland. Phylogenetic analysis of the E1A, fiber, and hexon genes revealed that isolates from Canada, the United States, and Ireland clustered together. Restriction enzyme analysis was not performed on the 3 isolates from Canada; however, phylogenetic analysis and the presence of the 6-nt deletion suggested that these isolates corresponded to the genomic variant HAdV-14p1.

## Conclusions

Our findings confirm the presence of HAdV-14 in Canada since the emergence of this serotype in North America was first described. In the case we describe, the patient sought medical care at our hospital after being ill for 3 days. She received a diagnosis of community-acquired pneumonia, was hospitalized, and died 6 days later. The patient maintained a normal level of total leukocytes during her illness but was lymphocytopenic. It has been reported that lymphocytopenia could be more common among severely ill patients with adenovirus infection (*5*). Epidemiologic data obtained from the patient’s family showed that she had not traveled outside New Brunswick in the 14 days preceding hospitalization, and she frequently participated in social gatherings. Transmission appeared to occur through close, person-to-person contact and among members of tight social networks, similar to transmission during an outbreak of HAdV-14 in Alaska (*1*). Family members also reported that in the weeks following the patient’s illness, many aboriginal members of the community had influenza-like symptoms, which could represent other unconfirmed cases of HAdV-14 infection. 

Epidemiologic data is lacking for the HAdV-14 variant emerging in Canada. Because clinicians do not always perform diagnostic tests for respiratory illnesses, cases of HAdV-14 infection might be diagnosed simply as acute respiratory illnesses or as unspecified sporadic viral pneumonias. During a 2008–2009 outbreak of adenovirus type 3 in New Brunswick, the same province in which the current study patient lived, we serotyped 17 strains and found no evidence of HAdV-14 (*10*). Moreover, a large 2007 Canadian study in the Toronto region failed to find any HAdV-14 strains among the 200 strains that were submitted; circulating HAdV strains were largely subtypes 1, 2, and 3 (*11*).

After finding these 3 cases, we notified public health authorities and the provincial Centre for Disease Control, who, in turn, informed physicians of the presence of HAdV-14 in New Brunswick. No other cases have been reported.
